# Influence of Olive Pomace Blending on Antioxidant Activity: Additive, Synergistic, and Antagonistic Effects

**DOI:** 10.3390/molecules26010169

**Published:** 2020-12-31

**Authors:** M. Antónia Nunes, Filip Reszczyński, Ricardo N. M. J. Páscoa, Anabela S. G. Costa, Rita C. Alves, Maria Beatriz P. P. Oliveira

**Affiliations:** REQUIMTE/LAQV, Department of Chemical Sciences, Faculty of Pharmacy of University of Porto, R. Jorge Viterbo Ferreira n°. 228, 4050-313 Porto, Portugal; antonianunes.maria@gmail.com (M.A.N.); f.reszczynski@gmail.com (F.R.); rnpascoa@ff.up.pt (R.N.M.J.P.); acosta@ff.up.pt (A.S.G.C.); beatoliv@ff.up.pt (M.B.P.P.O.)

**Keywords:** by-products, antioxidants, mixture, synergism, antagonism, additive

## Abstract

Food innovation is moving rapidly and comprises new categories of food products and/or ingredients with a natural and ecological origin. Monocultivar olive pomaces, individually or combined, can be a source of natural bioactive compounds suitable for food or cosmetic applications. This work aimed to assess the phenolics content and antioxidant activity of four monocultivar olive pomaces (Arbosana, Koroneiki, Oliana, and Arbequina) and forty-nine blends prepared with different proportions of each. Additive, synergistic, and antagonistic effects were studied. Among the monocultivar pomaces, Koroneiki and Arbosana were the richest in total phenolics (~15 mg gallic acid eq./g). Most of the interactions found in the blends were additive or synergistic, while very few antagonistic effects were observed. The best results were obtained for those blends where the Koroneiki variety predominated: (i) 90% Koroneiki, 4.75% Oliana, 3.75% Arbequina, 1.5% Arbosana; (ii) 65% Koroneiki, 29% Oliana, 3.25% Arbequina, 2.75% Arbosana; and (iii) 85% Koroneiki, 8.75% Arbequina, 3.5% Arbosana, 2.75% Oliana. In sum, these combinations can be advantageous in comparison to the individual use of monocultivar pomaces, presenting a higher potential to be used as functional ingredients or for bioactive compounds extraction, having in view the obtention of natural preservatives or food/cosmetic formula enhancers.

## 1. Introduction

In recent years, science and socio-economic players have been exploring sustainable solutions for agriculture. Ag-tech systems have been emerging to increase the production of several crops, in line with the belief that the current agro-food revolution must be complemented by ecological practices, such as the management of by-products [1]. The development of new products using natural compounds recovered from food by-products is a challenge and an attempt to meet the consumers’ current demand for natural and ecological products/ingredients. At the same time, it reduces the environmental and economic burden related to by-products management [2,3,4].

Considering the olive oil sector, the European Union produces 91% (14 million tonnes) of the world’s olive oil, and the Mediterranean region contributes to ≈ 70% of that amount [5]. In recent decades, there was a global intensification of olive oil production, particularly in some countries like Portugal, due to the innovation of the sector, new plantations, and national and European investments [6]. Along with the increasing production of olive oil, the disposal of raised amounts of several by-products (such as leaves, olive mill wastewaters, and olive pomace) is also a reality.

Nowadays, monocultivar olive oils are increasingly present on the market, but some producers also develop olive oil blends based on the chemical and organoleptic features of individual olive varieties [7]. For example, Arbequina, Arbosana, and Koroneiki are predominant cultivars in super-high-density orchards [8]. The Spanish Arbosana and Arbequina cultivars are mostly known for their precocity and high yield of fruits, and their olives have similar characteristics [9]. With a Greek origin, Koroneiki is very resistant to environmental conditions, as water stress and wind, but sensitive to infestation. The fruit is very hard to harvest due to its small size. However, its oil is very stable [10]. The Oliana cultivar was introduced very recently in olive orchards as a result of genetic crossing of Arbequina and Arbosana. Its most important feature is the minor size when compared with the parental trees and the larger productivity that facilitates the modern harvest techniques [11].

Olive pomace is a major output of olive oil production. The oil itself constitutes, depending on the selected cultivar and the extraction procedure, about 15–20% of the fruit [12,13]. The remaining part constitutes the olive pomace. This by-product contains, among other components, water, polysaccharides, minerals, fatty acids, and phenolic compounds [14], retaining around 98% of the polyphenols originally present in the fruit [15]. The total amount and profile of these compounds change according to the periods of growth, maturation, and ripening of the olive. Moreover, the intensity of phenolics biosynthesis is inversely correlated with the water availability in the soil where the tree grows. Indeed, the total content of phenolics in irrigated olive trees can be lower than in rain fed trees, and the profile can also differ [16,17]. Additionally, the olive oil processing techniques such as crushing, malaxation, and centrifugation can lead to a modification of the phenolics profile of olive oil and pomace [17].

Olive pomace antioxidants are regarded as cost-effective and a green alternative to currently used food preservatives. They can also be applied as enhancers of the nutritional profile of foodstuffs [18,19]. The main aim of this work was to ascertain the effect of blending monocultivar olive pomaces on the antioxidant properties of the samples. For that, the phenolics content and antioxidant activity of four monocultivar olive pomaces (Arbosana, Koroneiki, Oliana, and Arbequina) were explored for the first time, as well as the additive synergistic, and antagonistic effects occurring when different olive pomaces are blended in different proportions.

## 2. Results and Discussion

The phenolics found in olive pomace are a complex combination of compounds from different groups but connected by similarities such as chemical structure and biological properties. Some of the major compounds are (i) hydroxytyrosol, tyrosol and derivatives, (ii) iridoid precursors, (iii) secoiridoids (oleuropein, oleuropein aglycone, ligstroside, verbascoside) their derivatives), (iv) flavonoids (luteolin, apigenin, rutin and derivatives), (v) lignans (pinoresinol and derivatives), and (vi) phenolic acids (gallic acid, caffeic acid, cinnamic acid, p-coumaric acid, ferulic acid, vanillic acid, and shikimic acid) [19,20].

Hydroxytyrosol is one of the most relevant antioxidants of olive pomace. Its antioxidant activity is related to its ortho-diphenol moiety which acts as chain breaker by donating a hydrogen atom to peroxyl-radicals [21]. In this way, it contributes to the prevention of oxidative stress and related physiological and pathological processes (e.g., ageing, cancer, neurodegenerative and cardiovascular diseases). Hydroxytyrosol is also involved in the modulation of inflammatory response and insulin resistance [22], as well as in the activation of different cellular signaling pathways, increasing the endogenous defense systems against an oxidative stress state [23]. In general, the above-mentioned phenolics are multifunctional, which means that they can exert several antioxidant actions such as: (i) scavenging free radicals; (ii) inactivating metal catalysts; (iii) reducing hydroperoxides into stable hydroxyl derivatives; (iv) interacting with other reducing compounds in a synergistic way [24].

Table 1 shows the total phenolic content and the antioxidant activity of both monocultivar olive pomaces and different blends prepared with variable proportions of each monocultivar.

Considering the monocultivar samples, Koroneiki showed the highest total phenolics content (15.4 mg GAE/g), whereas Arbosana, Arbequina, and Oliana presented lower values, by this order: 14.7, 10.7, and 8.7 mg GAE/g. Concerning the antioxidant activity, Koroneiki achieved the highest values assessed by Ferric Reducing Antioxidant Power (FRAP) and DPPH^•^ inhibition assays (58.4 mg FSE/g and 20.0 mg TE/g, respectively), although no significant differences (*p* > 0.05) were observed in this last assay between Koroneiki and Arbosana (19.4 mg TE/g), the two varieties with the highest phenolics contents. Regarding the FRAP assay, no significant differences (*p* > 0.05) were observed between Oliana (27.9 mg FSE/g) and Arbequina (32.0 mg FSE/g) samples (Table 1).

It is known that the phenolic content of olives differs not only according to the agronomic practices (cultivation, irrigation management, and olive maturation), soil, and edaphoclimatic conditions, but also according to genetics. These factors are reflected in the phenolics amount and profiles of olive fruits and olive oils obtained from different cultivars [25,26]. Consequently, it was also expected that olive pomaces had different compositions. For instance, Talhaoui and colleagues [25] studied the phenolic compounds of olives and extra-virgin olive oil of six different olive cultivars: Arbequina, Picual, Sikitita, Arbosana, Changlot Real, and Koroneiki. Additionally, these authors also investigated the phenolics transfer to the oil. The olives and the respective oils presented a total of 33 and 20 phenolic compounds, respectively. Regarding the fruits, Changlot Real presented the highest values in total phenolics, followed by Arbosana. In turn, the olive oil with a major phenolics content was Picual, followed by Koroneiki. Significant differences were observed among all cultivars and it was concluded that, in general, a low amount of phenolics are transferred from the fruits to the oils [25]. According to this, it is possible to infer that phenolics remain mainly in the olive oil processing by-products, such as olive pomace [27]. Moreover, the chemical compounds present in olives undergo through a cascade of transformations during processing due to, for example, the enzymatic activity of polyphenol oxidase and β-glucosidase during pressing and malaxation. These changes can be both qualitative and quantitative since they are reflected either in the phenolic profile or in the total amount of the compounds of olive fruits and oils. Thus, similar variations in olive pomace phenolics are also expected [25].

In relation to the blends prepared in this study (Table 1), those containing a higher Koroneiki percentage (40, 50, and 60%) demonstrated greater antioxidant properties. Also, the blends with higher proportions of Koroneiki and Arbosana presented higher amounts of total phenolics. Indeed, the samples 18 (50% Koroneiki, 41.25% Arbequina, 5% Arbosana, 3.75% Oliana), 22 (90% Koroneiki, 4.75% Oliana, 3.75% Arbequina, 1.5% Arbosana), 41 (65% Koroneiki, 29% Oliana, 3.25% Arbequina, 2.75% Arbosana), 44 (85% Arbosana, 6.5% Koroneiki, 5.25% Arbequina, 3.25% Oliana), and 45 (85% Koroneiki, 8.75% Arbequina, 3.5% Arbosana, 2.75% Oliana) presented 18.1, 20.1, 21.2, 18.5, and 18.6 mg GAE/g of olive pomace, respectively, significantly higher values compared to the remaining blends. However, the antioxidant activity evaluated through the FRAP assay showed no significant differences between those samples and samples 25 (42% Koroneiki, 30% Oliana, 26% Arbequina, 2% Arbosana), 34 (55% Koroneiki, 30% Arbequina, 8.5% Oliana, 6.5% Arbosana), and 53 (48% Arbosana, 26% Koroneiki, 15% Arbequina, 11% Oliana). In fact, among all the mentioned samples, sample 45 presented the highest FRAP value (59.7 mg FSE/g) and sample 18 the lowest one (46.7 mg FSE/g). Regarding DPPH^•^ inhibition, the samples 34 (55% Koroneiki, 30% Arbequina, 8.5% Oliana, 6.5% Arbosana) and 41 (65% Koroneiki, 29% Oliana, 3.25% Arbequina, 2.75% Arbosana) presented the highest value: 21.5 and 21.4 mg TE/g, respectively.

The obtained results lead to questioning if there were in vitro additive, synergistic, or antagonistic interactions within the olive pomace blends, since all phytochemicals may interact with each other, influencing the total phenolics content of the samples as well as their antioxidant capacity [28]. Indeed, the combination of different compounds or distinct amounts of those compounds can lead to additive and synergistic effects or, in turn, to an antagonistic behavior. In the case of olive oil, synergistic effects between tyrosol, hydrocarbons (squalene) and phytosterols (β-sitosterol) have already been reported [29,30].

The complexity related to the type, amount, and bioactive action of phenolics in each monovarietal olive pomace leads to a multiplicity of interactions that are not limited to phenolic–phenolic ones but also includes other chemical components such as proteins, carbohydrates, fiber, fat and carotenoids [30]. Indeed, it is important to notice that each monovarietal pomace, individually, already presents a multitude of bioactive compounds trapped in a complex net of interactions, which can globally grow when interacting with other pomaces. By this way, in a broad sense, the function “interaction”, in this work, indicates a dynamic relationship among the bioactive compounds, particularly of phenolics, among the four olive pomaces.

Table 1 describes the type of interaction observed for each blend based on the predicted results calculated using the experimental values obtained for each monocultivar pomace. Although several factors can affect the antioxidant properties of a single compound, namely its concentration and the interaction with other constituents, the specific interactions that are known to occur are still not completely understood [31,32]. The complexity of reactions increases as many compounds are present in a sample and with the number of matrices combined, since this raises the amplitude of interactions [32]. Furthermore, two compounds that interact in one way in a reaction environment can exhibit other behaviors when exposed to another mixture with different constituents [33]. An antagonism occurs when two or more compounds react, and the result is lower than the mathematical sum of the individual effects [32]. Some of the studied blends showed antagonistic interactions, as described in Table 1.

The different interactions observed when mixing the blends can be related to several compounds. Despite the blends being composed of four different olive varieties, the amount of phenolics is alike in Arbosana and Koroneiki pomaces. Additionally, their profile in the monocultivar samples is expected to be similar: hydroxytyrosol is the major phenolic described in olive pomaces, followed mostly by tyrosol, which has a lower antioxidant activity in comparison to the former [34]. However, besides phenolics, several other compounds are present in olive pomaces that can also contribute to their antioxidant activity. The lipid fraction, for example, contains α-tocopherol that has strong antioxidant properties, known as the most potent radical-scavenging lipophilic antioxidant. In a previous study, we found that Arbequina, Arbosana, and Oliana pomaces contained higher amounts of α-tocopherol compared to the Koroneiki one [35].

In complex mixtures, the antagonistic effects can be observed when: (i) an efficient compound regenerates another one with lower antioxidant activity; (ii) complexes and adducts are formed between antioxidant compounds; (iii) antioxidant polymerization (mainly polyphenols) occurs during oxidation, which leads to the reduction in the antioxidant capacity [32,36]. In turn, the regeneration of powerful antioxidants by weaker ones results in synergism. Other mechanisms can be related to synergism, namely (i) the development of stable complexes among compounds with great antioxidant power; (ii) the formation of dimers and adducts and/or new phenolic products with a higher antioxidant capacity than the primary compounds. The additive effect may indicate lack of interactions during the radical neutralization. This means that each antioxidant is acting independently of the others present in a complex mixture [32].

When comparing the results obtained for the different blends (Table 1), few significant differences were observed among the samples regarding not only the total phenolics content, but also the antioxidant activities evaluated by both assays. This can be related to the low percentage variation between some blends that could be insufficient to cause significant differences in those parameters. However, the broad range of percentages used in this study allowed to foresee, at a large scale, the antioxidant yield of the mixtures.

The higher synergistic effect in phenolics content corresponded to the pomace blends containing Koroneiki (samples 18, 22, 41, and 45) and Arbosana (sample 44) in percentages above 50 and 85%, respectively. However, this was not directly correlated with the FRAP interactions (in which higher values were related to higher Arbosana percentages) or even with DPPH^•^ inhibition. This can be associated with the presence of several compounds that are contributing to the antioxidant activity and the different mechanisms of reaction of each method. While in the FRAP assay, the detected antioxidants are able to transfer an electron and reduce the 2,4,6-tripyridyl-s-triazine/ferric ion complex (antioxidants with a redox potential lower than 0.7 V), the DPPH^•^ inhibition method evaluates the ability of antioxidants to scavenge this radical, which can occur via both electron or H atom transfer (reduction and radical quenching, respectively). These two methods were selected to perform this study, since they have complementary mechanisms of action, which is advisable to better understand the antioxidant properties of the samples [37].

Most of the interactions among the four olive pomaces were synergistic regarding total phenolics (32 samples) and FRAP values (28 samples), and additive for DPPH^•^ inhibition (28 samples). In general, very few antagonistic interactions were observed (samples 12, 13, and 14, for total phenolics; 5 and 6, for FRAP; and 7 and 29, for DPPH^•^ inhibition). Sometimes, the same blend showed a synergism in phenolics content and an antagonism in the FRAP assay (e.g., samples 20 and 21); or an additive effect in phenolics, together with synergism in FRAP and antagonism in DPPH^•^ inhibition (sample 29). In this case, the major olive pomace present was Oliana (70%). This olive variety is not extensively characterized since it is recent in orchards, but it will be relevant to investigate its individual compounds and/or amounts that can differentiate its behavior from the other pomaces. Sample 6 presented antagonistic effects in FRAP and DPPH^•^ assays and an additive interaction in phenolics although, in this case, Arbequina (55%) and Arbosana (20%) were the major pomaces present, followed by Oliana (17.5%).

From all the blends, samples 22, 41, and 45 can be highlighted as they presented higher amounts of total phenolics and higher antioxidant activity. These samples are mostly composed of the Koroneiki olive pomace, the monocultivar with a higher amount of total phenolics, followed by Oliana (samples 22 and 41) and Arbequina (sample 45). All three blends presented synergistic (total phenolics) and additive (FRAP assay) effects. Concerning DPPH^•^ inhibition, samples 22 and 45 presented an additive effect, whereas in sample 41 a synergistic effect occurred.

In sum, the following combinations of pomace mixtures: (i) 90% Koroneiki, 4.75% Oliana, 3.75% Arbequina, 1.5% Arbosana; (ii) 65% Koroneiki, 29% Oliana, 3.25% Arbequina, 2.75% Arbosana; and (iii) 85% Koroneiki, 8.75% Arbequina, 3.5% Arbosana, 2.75% Oliana, presented major potential to be used as functional ingredients or for bioactive compounds extraction since their antioxidant properties are increased. Those can be applied, for example, as natural preservatives and/or food and cosmetics formula enhancers.

## 3. Materials and Methods

### 3.1. Reagents and Standards

Absolute ethanol and sodium carbonate decahydrate were purchased from Merck (Darmstadt, Germany). Gallic acid, heptahydrate ferrous sulfate, trolox, Folin-Ciocalteu reagent, 2,2-diphenyl-1-picrylhydrazyl radical (DPPH^•^), ferric chloride, 2,4,6-tripyridyl-triazine, sodium acetate were all acquired from Sigma-Aldrich (St. Louis, MO, USA). All other reagents were of analytical grade.

### 3.2. Samples

Olive pomace samples (≈10 kg) obtained from a two-phase extraction olive mill (in Alentejo, Portugal) were kindly provided by a national company (SOVENA) in the season 2017/2018 (January 2018). Four monocultivar olive pomaces were collected after individual olive oil processing: Arbosana, Koroneiki, Oliana, and Arbequina. As soon as samples arrived at the laboratory, they were freeze-dried and ground (GM200 Grindomix, Retch, Germany). Then, the four olive pomaces were blended to obtain the 49 mixtures described in Table 1. The mixtures were prepared following a D-optimal mixture experimental design with extra samples carried out in the Design Expert software (Stat-Ease Inc., MN, USA).

### 3.3. Antioxidants Extraction Procedure

The extraction procedure was performed using an ethanol:water solution (80/20; *v/v*) with magnetic stirring (Horizontal Shaker KS 15 B, Edmund Bühler GmbH, Germany) at room temperature for 3 h. Then, samples were filtered (Whatman n.° 4 filter) and the extracts analyzed. Extractions were performed in triplicate.

### 3.4. Total Phenolics Content

The determination of total phenolics content was carried out, in triplicate, as described by Costa et al. [37] with some modifications. Briefly, 30 µL of extract was mixed with 150 µL of Folin–Ciocalteu reagent (10%) and 120 μL of Na_2_CO_3_ solution (7.5%) in a 96-well microplate. The solution was incubated at 45 °C, for 15 min, in the dark. The absorbance was measured at 765 nm using a Synergy HT Microplate Reader (BioTek Instruments, Inc., Winooski, VT, USA). The results were expressed as mg of gallic acid equivalents (GAE)/g of olive pomace (dry weight).

### 3.5. Antioxidant Activity

The antioxidant activity was evaluated by two complementary methods: ferric reducing antioxidant power (FRAP) and the DPPH^•^ scavenging ability. Each assay was performed in triplicate.

#### 3.5.1. FRAP

For the FRAP assay, an aliquot (35 μL) of each sample was mixed with 265 µL of FRAP reagent prepared according to Costa et al. [37]. Then, the mixture was incubated for 30 min at 37 °C (protected from light). Measurement of absorbance was performed at 595 nm in a Synergy HT Microplate Reader (BioTek Instruments, Inc., Winooski, VT, USA). The results were presented as mg ferrous sulfate equivalents (FSE)/g of olive pomace (dry weight).

#### 3.5.2. DPPH^•^ Scavenging Ability

The anti-radical ability of the extracts was assessed according to Costa et al. [37]. Briefly, an aliquot (30 µL) was mixed with 270 µL of an ethanolic DPPH^•^ solution (6.0 × 10^−5^ mol/L). The reaction kinetic was monitored (with 2 min intervals) at 525 nm (Synergy HT Microplate Reader, BioTek Instruments, Inc., Winooski, VT, USA) until a plateau was attained. The results were expressed as mg of trolox equivalents (TE)/g of olive pomace (dry weight).

### 3.6. Synergistic, Additive, and Antagonistic Interactions

The in vitro synergistic, additive, and antagonistic interactions obtained with olive pomaces blending were studied.

Predicted values were calculated through the mathematical sum of the total phenolic contents and antioxidant capacity values obtained for the monocultivar olive pomaces.

Experimental values determined for the blended samples were compared with the respective predicted ones. When significant differences (*p* < 0.05) were found between experimental and predicted data, the effects were classified as synergistic or antagonistic (for positive or negative differences, respectively). When no significant differences (*p* > 0.05) were observed between experimental and predicted data, the effect was classified as additive.

### 3.7. Statistical Analysis

Statistical analysis was performed using IBM SPSS v. 25 (IBM Corp., Armonk, NY, USA). Data are expressed as mean ± standard deviation (*n* = 9). The Shapiro–Wilk test was used to evaluate data normality. The one-way ANOVA was used to assess significant differences between samples, followed by Tukey’s HSD or Dunett T3 post hoc test (selected based on the equality of the variances) to make pairwise comparisons between means. Student’s *t*-test was used to define the type of effect (synergistic, antagonistic, or additive) by comparing the expected and the experimental values. The level of significance for all hypothesis tests (*p*) was 0.05.

## 4. Conclusions

Olive oil is mostly obtained from different mixed olive cultivars, usually present in the same orchard, but the market demand for monocultivar olive oils has been increasing. Huge amounts of olive pomace are being produced and suggestions on how it can be valued and recovered are needed.

The present work, besides anticipating a valuable application of bioactive compounds derived from Arbosana, Koroneiki, Oliana, and Arbequina cultivars as natural antioxidants, also shows that it can be gainful to mix the monocultivar pomaces rather than use them individually. The key is to select the most promising varieties and the adequate proportions to obtain the greatest synergistic effect among the samples. In this work, the best results were obtained using a mixture where the Koroneiki variety predominates.

Nevertheless, further studies are needed to explore the association of the described interactions and the phenolics profile. Characteristic individual olive pomace compounds and resultant interactions should also be investigated.

## Figures and Tables

**Table 1:** Total phenolics contents and antioxidant activity of monocultivar olive pomaces and blends. Experimental, predicted results, and interactions.

Sample	Composition (Variety%)				Total Phenolics (mg GAE/g)				Ferric Reducing Antioxidant Power (mg FSE/g)				DPPH^•^ Scavenging Ability (mg TE/g)			
	Ar	K	Ol	Aq	Experimental	Predicted	*p*-Value	Interaction	Experimental	Predicted	*p*-Value	Interaction	Experimental	Predicted	*p*-Value	Interaction
1	100	0	0	0	14.7 ± 1.1 ^efghijkl^				48 ± 3.8 ^defghijk^				19.4 ± 1.2 ^abcdefg^			
2	0	100	0	0	15.4 ± 1.3 ^cdefghijk^				58.4 ± 10.1 ^ab^				20.0 ± 1.9 ^abcdef^			
3	0	0	100	0	8.7 ± 0.7 ^p^				27.9 ± 1.5 ^q^				12.5 ± 0.4 ^q^			
4	0	0	0	100	10.7 ± 0.8 ^o^				32.0 ± 0.3 ^pq^				15.1 ± 0.2 ^jklmnopq^			
5	10	12.5	32.5	45	10.8 ± 0.1 ^o^	11.0 ± 0.5	0.409	**Ad**	30.5 ± 0.3 ^pq^	35.6 ± 1.2	0.000	**At**	14.8 ± 0.1 ^klmnopq^	15.3 ± 0.3	0.124	**Ad**
6	20	7.5	17.5	55	10.7 ± 0.8 ^o^	11.5 ± 0.5	0.100	**Ad**	29 ± 3.8 ^q^	36.5 ± 1.3	0.000	**At**	14.1 ± 2.0 ^opq^	15.9 ± 0.5	0.024	**At**
7	30	17.5	45	7.5	12.6 ± 0.9 ^ijklmno^	11.8 ± 0.5	0.134	**Ad**	38.3 ± 3.3 ^nop^	39.6 ± 1.9	0.365	**Ad**	17.6 ± 1.0 ^cdefghijklmno^	16.1 ± 0.7	0.009	**Sy**
8	40	32.5	25	2.5	13.1 ± 0.4 ^hijklmno^	13.3 ± 0.8	0.613	**Ad**	41.5 ± 4.3 ^klmno^	46.0 ± 3.5	0.051	**Ad**	20.5 ± 1.8 ^abcd^	17.8 ± 1.0	0.007	**Sy**
9	50	22	22.5	5.5	14.9 ± 1.1 ^defghijkl^	13.3 ± 0.9	0.047	**Sy**	44.5 ± 3.9 ^ijklmno^	44.9 ± 3.1	0.820	**Ad**	18.8 ± 0.2 ^abcdefghi^	17.8 ± 1.3	0.194	**Ad**
10	60	5.5	12	22.5	15.4 ± 1.0 ^cdefghijk^	13.1 ± 0.9	0.002	**Sy**	46.4 ± 3.1 ^ghijklm^	42.6 ± 2.3	0.030	**Sy**	18.3 ± 2.1 ^abcdefghijk^	17.7 ± 1.7	0.563	**Ad**
11	70	13.75	4	12.25	15.6 ± 1.2 ^cdefghi^	14.0 ± 1.1	0.045	**Sy**	43.5 ± 3.3 ^ijklmno^	46.7 ± 3.3	0.088	**Ad**	17.8 ± 1.1 ^bcdefghijklmn^	18.7 ± 2.0	0.435	**Ad**
12	80	2	13.5	4.5	10.6 ± 0.9 ^o^	13.7 ± 1.2	0.001	**At**	49.9 ± 7.5 ^cdefghij^	44.8 ± 2.6	0.083	**Ad**	16.7 ± 1.9 ^efghijklmnop^	18.3 ± 2.4	0.195	**Ad**
13	90	2.5	2.5	5	10.9 ± 0.2 ^o^	14.3 ± 1.4	0.000	**At**	46.8 ± 2.6 ^fghijklm^	47.0 ± 3.1	0.904	**Ad**	18 ± 1.3 ^abcdefghijkl^	19.1 ± 2.7	0.469	**Ad**
14	67.5	10	5.5	17	10.6 ± 0.7 ^o^	13.7 ± 1.0	0.000	**At**	50.6 ± 3.7 ^bcdefghi^	45.2 ± 2.9	0.016	**Sy**	19.1 ± 2.4 ^abcdefghi^	18.4 ± 1.9	0.509	**Ad**
15	55	19.75	18	7.25	12.7 ± 0.9 ^ijklmno^	13.4 ± 0.9	0.250	**Ad**	41.9 ± 2.3 ^jklmno^	45.3 ± 3.1	0.050	**Ad**	18.1 ± 1.7 ^abcdefghijkl^	18.0 ± 1.5	0.907	**Ad**
16	35	30	14.25	20.75	13.1 ± 0.2 ^hijklmno^	13.2 ± 0.7	0.707	**Ad**	46.0 ± 5.3 ^hijklmno^	44.9 ± 3.4	0.659	**Ad**	20.1 ± 1.4 ^abcdef^	17.7 ± 0.9	0.005	**Sy**
17	15	40	31	14	16.2 ± 0.1 ^cdefgh^	12.5 ± 0.5	0.000	**Sy**	47.6 ± 0.2 ^efghijk^	43.7 ± 3.4	0.043	**Sy**	18.8 ± 0.4 ^abcdefghi^	16.9 ± 0.7	0.001	**Sy**
18	5	50	3.75	41.25	18.1 ± 0.4 ^abcd^	13.2 ± 0.6	0.002	**Sy**	46.7 ± 0.7 ^fghijklm^	45.9 ± 4.3	0.680	**Ad**	17.9 ± 0.2 ^bcdefghijklm^	17.7 ± 1.0	0.839	**Ad**
19	12.5	60	16.5	11	17.8 ± 1.8 ^bcde^	13.7 ± 0.7	0.000	**Sy**	50.5 ± 0.1 ^bcdefghi^	49.2 ± 5.1	0.631	**Ad**	17.5 ± 1.7 ^cdefghijklmno^	18.2 ± 1.0	0.644	**Ad**
20	2.5	70	8.75	18.75	17.5 ± 0.1 ^bcdef^	13.9 ± 0.8	0.000	**Sy**	43.4 ± 5.4 ^ijklmno^	50.6 ± 5.7	0.043	**At**	18.3 ± 1.5 ^abcdefghijk^	18.4 ± 1.4	0.946	**Ad**
21	7.5	80	6	6.5	17.3 ± 1.8 ^bcdefg^	14.6 ± 1.0	0.007	**Sy**	41.8 ± 0.3 ^klmno^	54.1 ± 6.6	0.001	**At**	17.3 ± 0.9 ^cdefghijklmno^	19.2 ± 1.5	0.946	**Ad**
22	1.5	90	4.75	3.75	20.1 ± 0.8 ^ab^	14.9 ± 1.0	0.001	**Sy**	56.9 ± 6.3 ^abc^	55.8 ± 7.3	0.776	**Ad**	20.9 ± 1.3 ^abc^	19.4 ± 1.7	0.065	**Ad**
23	45	24.5	10	20.5	15.1 ± 1.3 ^defghijkl^	13.4 ± 0.8	0.026	**Sy**	41.8 ± 5.4 ^klmno^	45.3 ± 3.3	0.150	**Ad**	15.9 ± 0.5 ^ghijklmnopq^	18.0 ± 1.1	0.093	**Ad**
24	19	28	20	33	14.8 ± 2.2 ^efghijkl^	12.4 ± 0.5	0.030	**Sy**	43.4 ± 3.8 ^ijklmno^	41.6 ± 2.7	0.312	**Ad**	20.5 ± 1.0 ^abcd^	16.8 ± 0.5	0.000	**Sy**
25	2	42	30	26	16.2 ± 0.7 ^cdefgh^	12.1 ± 0.5	0.000	**Sy**	53.5 ± 0.6 ^abcdefgh^	42.2 ± 3.3	0.000	**Sy**	18.2 ± 0.1 ^abcdefghijk^	16.5 ± 0.9	0.008	**Sy**
26	9	20.5	40	30.5	13.5 ± 0.1 ^hijklmno^	11.2 ± 0.4	0.006	**Sy**	48.2 ± 2.0 ^defghijk^	37.2 ± 1.7	0.000	**Sy**	15.3 ± 0.2 ^jklmnopq^	15.5 ± 0.4	0.463	**Ad**
27	33.5	4	50	12.5	12.2 ± 0.4 ^klmno^	11.2 ± 0.5	0.025	**Sy**	47.5 ± 1 ^efghijkl^	36.4 ± 1.0	0.000	**Sy**	14.7 ± 0.3 ^lmnopq^	15.5 ± 0.9	0.106	**Ad**
28	4	9	60	27	10.9 ± 0.3 ^o^	10.1 ± 0.5	0.081	**Ad**	39.5 ± 2.5 ^lmno^	32.6 ± 0.6	0.000	**Sy**	12.7 ± 0.1 ^q^	14.2 ± 0.4	0.080	**Ad**
29	23	3.5	70	3.5	10.8 ± 0.5 ^o^	10.4 ± 0.5	0.371	**Ad**	44.9 ± 1.7 ^ijklmno^	33.7 ± 0.6	0.000	**Sy**	13.2 ± 0.4 ^pq^	14.5 ± 0.6	0.008	**At**
30	7	11	80	2	12.4 ± 0.3 ^jklmno^	9.9 ± 0.4	0.000	**Sy**	45.6 ± 1.5 ^hijklmno^	32.8 ± 0.6	0.000	**Sy**	14.3 ± 0.4 ^nopq^	13.9 ± 0.4	0.492	**Ad**
31	3	6	90	1	11.0 ± 0.1 ^no^	9.3 ± 0.5	0.001	**Sy**	43.3 ± 1.8 ^ijklmno^	30.4 ± 0.7	0.000	**Sy**	12.4 ± 0.3 ^q^	13.2 ± 0.5	0.235	**Ad**
32	13	33	44	10	12.3 ± 0.4 ^jklmno^	11.9 ± 0.4	0.327	**Ad**	42.7 ± 4.5 ^ijklmno^	41.0 ± 2.6	0.410	**Ad**	15.7 ± 0.3 ^ijklmnopq^	16.2 ± 0.6	0.314	**Ad**
33	25	3	52	20	13.1 ± 0.8 ^hijklmno^	10.8 ± 0.5	0.000	**Sy**	45.3 ± 1.7 ^ijklmno^	34.7 ± 0.7	0.000	**Sy**	17.1 ± 0.4 ^defghijklmno^	15.0 ± 0.6	0.005	**Sy**
34	6.5	55	8.5	30	17.1 ± 1.2 ^bcdefg^	13.4 ± 0.6	0.000	**Sy**	56.1 ± 6.0 ^abcd^	47.2 ± 4.7	0.010	**Sy**	21.5 ± 0.8 ^a^	17.9 ± 1.1	0.000	**Sy**
35	12	16	32	40	11.9 ± 1.0 ^lmno^	11.3 ± 0.4	0.285	**Ad**	43.7 ± 2.9 ^ijklmno^	36.8 ± 1.5	0.001	**Sy**	16.6 ± 1.2 ^fghijklmnopq^	15.6 ± 0.4	0.084	**Ad**
36	23.5	15	11.5	50	14.2 ± 0.1 ^ghijklmn^	12.1 ± 0.5	0.005	**Sy**	48.4 ± 1.1 ^defghijk^	39.3 ± 2.0	0.000	**Sy**	18.4 ± 0.8 ^abcdefghijk^	16.6 ± 0.5	0.002	**Sy**
37	16.5	10.5	13	60	15.2 ± 1.1 ^defghijk^	11.6 ± 0.5	0.000	**Sy**	48.4 ± 0.7 ^defghijk^	36.9 ± 1.5	0.000	**Sy**	17.9 ± 0.9 ^abcdefghijkl^	16.0 ± 0.4	0.045	**Sy**
38	4.5	21	4.5	70	13.6 ± 0.4 ^hijklmno^	11.8 ± 0.6	0.000	**Sy**	46.3 ± 0.4 ^ghijklmn^	38.1 ± 2.2	0.000	**Sy**	18.2 ± 0.5 ^abcdefghijk^	16.2 ± 0.6	0.008	**Sy**
39	6	5	9	80	14.6 ± 0.5 ^efghijkl^	11.0 ± 0.7	0.000	**Sy**	48.5 ± 0.5 ^defghijk^	33.9 ± 1.1	0.000	**Sy**	18.5 ± 0.1 ^abcdefghij^	15.4 ± 0.4	0.000	**Sy**
40	1	8	1	90	13.4 ± 0.2 ^hijklmno^	11.1 ± 0.8	0.004	**Sy**	45.3 ± 2.4 ^ijklmno^	34.2 ± 1.4	0.000	**Sy**	18.9 ± 1.5 ^abcdefghi^	15.5 ± 0.5	0.000	**Sy**
41	2.75	65	29	3.25	21.2 ± 0.9 ^a^	13.3 ± 0.7	0.000	**Sy**	54.9 ± 4.7 ^abcde^	48.4 ± 5.0	0.070	**Ad**	21.4 ± 1.0 ^ab^	17.7 ± 1.2	0.000	**Sy**
42	11	19	63	7	13.6 ± 1.0 ^hijklmno^	10.8 ± 0.3	0.000	**Sy**	41.5 ± 0.6 ^klmno^	36.2 ± 1.3	0.000	**Sy**	15.1 ± 0.6 ^jklmnopq^	14.9 ± 0.4	0.652	**Ad**
43	5.5	4.5	27.5	62.5	11.1 ± 0.2 ^mno^	10.6 ± 0.6	0.321	**Ad**	39.0 ± 1.6 ^mno^	33.0 ± 0.9	0.000	**Sy**	15 ± 0.3 ^jklmnopq^	14.9 ± 0.3	0.707	**Ad**
44	85	6.5	3.25	5.25	18.5 ± 0.6 ^abc^	14.3 ± 1.3	0.001	**Sy**	54.3 ± 0.3 ^abcdefg^	47.2 ± 3.2	0.001	**Sy**	19.4 ± 1.0 ^abcdefgh^	19.0 ± 2.5	0.784	**Ad**
45	3.5	85	2.75	8.75	18.6 ± 0.9 ^abc^	14.8 ± 1.0	0.002	**Sy**	59.7 ± 5.7 ^a^	54.9 ± 7.0	0.189	**Ad**	19.9 ± 1.3 ^abcdef^	19.3 ± 1.6	0.534	**Ad**
46	5.75	1.25	85	8	10.9 ± 0.5 ^no^	9.3 ± 0.5	0.004	**Sy**	38.2 ± 3.1 ^op^	29.8 ± 0.9	0.000	**Sy**	14.2 ± 1.0 ^mnopq^	13.2 ± 0.4	0.072	**Ad**
47	7.25	0.75	7	85	13.8 ± 0.1 ^hijklmno^	10.9 ± 0.7	0.001	**Sy**	46.4 ± 1.8 ^ghijklm^	33.1 ± 1.0	0.000	**Sy**	19.0 ± 1.4 ^abcdefghi^	15.3 ± 0.3	0.000	**Sy**
48	21	23	27	29	15.5 ± 1.2 ^cdefghij^	12.1 ± 0.5	0.001	**Sy**	47.5 ± 0.1 ^efghijkl^	40.3 ± 2.3	0.001	**Sy**	20.2 ± 0.3 ^abcde^	16.5 ± 0.5	0.000	**Sy**
49	18	36	21	25	14.3 ± 0.3 ^fghijklm^	12.7 ± 0.5	0.025	**Sy**	46.9 ± 1.2 ^efghijklm^	43.5 ± 3.3	0.083	**Ad**	17.9 ± 0.9 ^abcdefghijkl^	17.1 ± 0.6	0.264	**Ad**
50	1.25	26.75	35	37	12.7 ± 1.0 ^ijklmno^	11.3 ± 0.5	0.052	**Ad**	41.6 ± 2.6 ^klmno^	37.8 ± 2.0	0.016	**Sy**	15.8 ± 0.9 ^hijklmnopq^	15.6 ± 0.7	0.755	**Ad**
51	27	11.75	15	46.25	12.4 ± 1.0 ^ijklmno^	12.0 ± 0.5	0.335	**Ad**	47.7 ± 3 ^efghijk^	38.8 ± 1.8	0.000	**Sy**	17.5 ± 0.1 ^cdefghijklmno^	16.5 ± 0.6	0.006	**Sy**
52	16.75	16.75	37	29.5	13.6 ± 0.1 ^hijklmno^	11.4 ± 0.4	0.008	**Sy**	46.3 ± 2.8 ^ghijklmn^	37.6 ± 1.6	0.001	**Sy**	17.1 ± 1.5 ^defghijklmno^	15.7 ± 0.4	0.04	**Sy**
53	48	26	11	15	14.4 ± 2.2 ^fghijkl^	13.6 ± 0.8	0.420	**Ad**	54.7 ± 4.6 ^abcdef^	46.1 ± 3.5	0.004	**Sy**	19.9 ± 0.8 ^abcdef^	18.2 ± 1.2	0.035	**Sy**

Data are expressed as mean ± standard deviation. ^a–q^, Different letters within each “Experimental” column represent significant statistical differences between samples (1–53), at *p* < 0.05. When significant differences (*p* < 0.05) were found between experimental and predicted data, the effects were classified as synergistic or antagonistic (for positive or negative differences, respectively). When no significant differences (*p* > 0.05) were observed between experimental and predicted data, the effect was classified as additive. Ar, Arbosana; K, Koroneiki; Ol, Oliana; Aq, Arbequina; Ad, Additive; Sy, Synergistic; At, Antagonistic; GAE, Gallic acid equivalents; FSE, ferrous sulfate equivalents; TE—Trolox equivalents.

## Data Availability

Not applicable.

## References

[1] Rotz S., Duncan E., Small M., Botschner J., Dara R., Mosby I., Reed M., Fraser E.D.G. (2019). The politics of digital agricultural technologies: A preliminary review. Sociol. Rural..

[2] Verain M.C.D., Sijtsema S.J., Antonides G. (2016). Consumer segmentation based on food-category attribute importance: The relation with healthiness and sustainability perceptions. Food Qual. Prefer..

[3] Aschemann-Witzel J., Varela P., Peschel A.O. (2019). Consumers’ categorization of food ingredients: Do consumers perceive them as ‘clean label’ producers expect? An exploration with projective mapping. Food Qual. Prefer..

[4] Angus A., Westbrook G. (2020). Top 10 Global Consumer Trends 2020.

[5] Sánchez de la Campa A.M., Salvador P., Fernández-Camacho R., Artiñano B., Coz E., Márquez G., Sánchez-Rodas D., de la Rosa J. (2018). Characterization of biomass burning from olive grove areas: A major source of organic aerosol in PM10 of Southwest Europe. Atmos. Res..

[6] Neves B., Pires I.M. (2018). The Mediterranean diet and the increasing demand of the olive oil sector: Shifts and environmental consequences. REGION.

[7] Pereira C., Costa Freitas A.M., Cabrita M.J., Garcia R. (2020). Assessing tyrosol and hydroxytyrosol in Portuguese monovarietal olive oils: Revealing the nutraceutical potential by a combined spectroscopic and chromatographic techniques-based approach. LWT Food Sci. Technol..

[8] Connor D.J., Gómez-del-Campo M., Rousseaux M.C., Searles P.S. (2014). Structure, management and productivity of hedgerow olive orchards: A review. Sci. Hortic..

[9] Vossen P. (2007). Olive oil: History, production, and characteristics of the world’s classic oils. HortScience.

[10] Dabbou S., Dabbou S., Chehab H., Brahmi F., Taticchi A., Servili M., Hammami M. (2011). Chemical composition of virgin olive oils from Koroneiki cultivar grown in Tunisia with regard to fruit ripening and irrigation regimes. Int. J. Food Sci. Technol..

[11] Cunill M., Salvador D. (2014). Oliana: Una nueva variedad de olivo adaptada al sistema superintensivo. Olint Plantas Olivo Mag..

[12] Chanioti S., Tzia C. (2019). Evaluation of ultrasound assisted and conventional methods for production of olive pomace oil enriched in sterols and squalene. LWT Food Sci. Technol..

[13] Di Giovacchino L., Preziuso S.M., Di Serio M.G., Mucciarella M.R., Di Loreto G., Lanza B. (2017). Double extraction of olive oil in large oil mills of Southern Italy: Effects on extraction efficiency, oil quality, and economy of the process. Eur. J. Lipid Sci. Technol..

[14] Rodrigues F., Nunes M.A., Oliveira M.B.P.P., Galanakis C.M. (2017). Chapter 12—Applications of recovered bioactive compounds in cosmetics and health care products. Olive Mill Waste.

[15] Chanioti S., Tzia C. (2017). Optimization of ultrasound-assisted extraction of oil from olive pomace using response surface technology: Oil recovery, unsaponifiable matter, total phenol content and antioxidant activity. LWT Food Sci. Technol..

[16] Jiménez-Herrera R., Pacheco-López B., Peragón J. (2019). Water stress, irrigation and concentrations of pentacyclic triterpenes and phenols in *Olea europaea* L. cv. Picual Olive Trees. Antioxidants.

[17] Servili M., Esposto S., Fabiani R., Urbani S., Taticchi A., Mariucci F., Selvaggini R., Montedoro G.F. (2009). Phenolic compounds in olive oil: Antioxidant, health and organoleptic activities according to their chemical structure. Inflammopharmacology.

[18] Nunes M.A., Pimentel F.B., Costa A.S.G., Alves R.C., Oliveira M.B.P.P. (2016). Olive by-products for functional and food applications: Challenging opportunities to face environmental constraints. Innov. Food Sci. Emerg. Technol..

[19] Peralbo-Molina Á., Priego-Capote F., Luque de Castro M.D. (2012). Tentative identification of phenolic compounds in olive pomace extracts using liquid chromatography–tandem mass spectrometry with a quadrupole–quadrupole-time-of-flight mass detector. J. Agric. Food Chem..

[20] Paze I.C., Lozano-Sánchez J., Borrás-Linares I., Nuñez H., Robert P., Segura-Carretero A. (2019). Obtaining an extract rich in phenolic compounds from olive pomace by pressurized liquid extraction. Molecules.

[21] Boronat A., Mateus J., Soldevila-Domenech N., Guerra M., Rodríguez-Morató J., Varon C., Muñoz D., Barbosa F., Morales J.C., Gaedigk A. (2019). Cardiovascular benefits of tyrosol and its endogenous conversion into hydroxytyrosol in humans. A randomized, controlled trial. Free Radic. Biol. Med..

[22] Čepo D.V., Radić K., Jurmanović S., Jug M., Grdić Rajković M., Pedisić S., Moslavac T., Albahari P. (2018). Valorization of olive pomace-based nutraceuticals as antioxidants in chemical, food, and biological models. Molecules.

[23] Marković A.K., Torić J., Barbarić M., Jakobušić Brala C. (2019). Hydroxytyrosol, tyrosol and derivatives and their potential effects on human health. Molecules.

[24] Frankel E.N., Finley J.W. (2008). How to standardize the multiplicity of methods to evaluate natural antioxidants. J. Agric. Food Chem..

[25] Talhaoui N., Gómez-Caravaca A.M., León L., De la Rosa R., Fernández-Gutiérrez A., Segura-Carretero A. (2016). From olive fruits to olive oil: Phenolic compound transfer in six different olive cultivars grown under the same agronomical conditions. Int. J. Mol. Sci..

[26] Vidal A.M., Alcalá S., De Torres A., Moya M., Espínola F. (2019). Characterization of olive oils from superintensive crops with different ripening degree, irrigation management, and cultivar: (Arbequina, Koroneiki, and Arbosana). Eur. J. Lipid Sci. Technol..

[27] Klen T.J., Vodopivec B.M. (2012). The fate of olive fruit phenols during commercial olive oil processing: Traditional press versus continuous two- and three-phase centrifuge. LWT Food Sci. Technol..

[28] Wang S., Meckling K.A., Marcone M.F., Kakuda Y., Tsao R. (2011). Synergistic, additive, and antagonistic effects of food mixtures on total antioxidant capacities. J. Agric. Food Chem..

[29] Moreno J.J. (2003). Effect of olive oil minor components on oxidative stress and arachidonic acid mobilization and metabolism by macrophages RAW 264.7. Free Radic. Biol. Med..

[30] Tresserra-Rimbau A., Lamuela-Raventos R.M., Moreno J.J. (2018). Polyphenols, food and pharma. Current knowledge and directions for future research. Biochem. Pharm..

[31] Giovagnoli-Vicuña C., Pizarro S., Briones-Labarca V., Delgadillo A. (2019). A square wave voltammetry study on the antioxidant interaction and effect of extraction method for binary fruit mixture extracts. J. Chem..

[32] Olszowy-Tomczyk M. (2020). Synergistic, antagonistic, and additive antioxidant effects in the binary mixtures. Phytochem. Rev..

[33] Palma A., Ruiz Montoya M., Díaz M.J., Arteaga J.F., Brito R.E., Mellado J.M.R. (2017). Evaluation of synergistic and antagonistic effects between some selected antioxidants by means of an electrochemical technique. Int. J. Food Sci. Technol..

[34] Nunes M.A., Costa A.S.G., Bessada S., Santos J., Puga H., Alves R.C., Freitas V., Oliveira M.B.P.P. (2018). Olive pomace as a valuable source of bioactive compounds: A study regarding its lipid- and water-soluble components. Sci. Total Env..

[35] Nunes M.A., Páscoa R.N.M.J., Alves R.C., Costa A.S.G., Bessada S., Oliveira M.B.P.P. (2020). Fourier transform near infrared spectroscopy as a tool to discriminate olive wastes: The case of monocultivar pomaces. Waste Manag..

[36] Hotta H., Sakamoto H., Nagano S., Osakai T., Tsujino Y. (2001). Unusually large numbers of electrons for the oxidation of polyphenolic antioxidants. Biochim. Biophys. Acta.

[37] Costa A.S.G., Alves R.C., Vinha A.F., Costa E., Costa C.S.G., Nunes M.A., Almeida A.A., Santos-Silva A., Oliveira M.B.P.P. (2018). Nutritional, chemical and antioxidant/pro-oxidant profiles of silverskin, a coffee roasting by-product. Food Chem..

